# Temporal dynamics of learning-promoted synaptic diversity in CA1 pyramidal neurons

**DOI:** 10.1096/fj.201801893RRR

**Published:** 2019-11-05

**Authors:** Yuya Sakimoto, Hiroyuki Kida, Dai Mitsushima

**Affiliations:** * Department of Physiology, Graduate School of Medicine, Yamaguchi University, Ube, Japan;; † The Research Institute for Time Studies, Yamaguchi University, Yamaguchi, Japan

**Keywords:** AMPA receptor, glutamate, GABA_A_ receptor, GABA, contextual learning

## Abstract

Although contextual learning requires plasticity at both excitatory and inhibitory (*E*/*I*) synapses in cornu ammonis 1 (CA1) neurons, the temporal dynamics across the neuronal population are poorly understood. Using an inhibitory avoidance task, we analyzed the dynamic changes in learning-induced *E*/*I* synaptic plasticity. The training strengthened GABA_A_ receptor–mediated synapses within 1 min, peaked at 10 min, and lasted for over 60 min. The intracellular loop (Ser^408−409^) of GABA_A_ receptor β_3_ subunit was also phosphorylated within 1 min of training. As the results of strengthening of α-amino-3-hydroxyl-5-methyl-4-isoxazole-propionate receptor–mediated synapses, CA1 pyramidal neurons exhibited broad diversity of *E*/*I* synaptic currents within 5 min. Moreover, presynaptic glutamate release probability at basal dendrites also increased within 5 min. To further quantify the diversified *E*/*I* synaptic currents, we calculated self-entropy (bit) for individual neurons. The neurons showed individual levels of the parameter, which rapidly increased within 1 min of training and maintained for over 60 min. These results suggest that learning-induced synaptic plasticity is critical immediately following encoding rather than during the retrieval phase of the learning. Understanding the temporal dynamics along with the quantification of synaptic diversity would be necessary to identify a failure point for learning-promoted plasticity in cognitive disorders.—Sakimoto, Y., Kida, H., Mitsushima, D. Temporal dynamics of learning-promoted synaptic diversity in CA1 pyramidal neurons.

The hippocampus is a primary area for contextual memory (1), known to process spatio-temporal information (2, 3) within a specific episode (4). Long-term strengthening of glutamatergic transmission has been identified as a mechanism of contextual learning in the dorsal cornu ammonis 1 (CA1) area of the hippocampus (5), and CA1-specific immobilization or blockade of α-amino-3-hydroxyl-5-methyl-4-isoxazole-propionate (AMPA) receptor delivery can impair the performance (6, 7), indicating a causal relationship between learning and the receptor delivery into the synapses.

AMPA receptors are tetrameric [glutamate receptor subunit A (GluA)1–4] (8, 9) ligand-gated cation channels at glutamatergic synapses. Considering that each presynaptic vesicle contains ∼2000 molecules of glutamate (10, 11), we quantified miniature postsynaptic AMPA receptor current induced by single-synaptic vesicle of glutamate [miniature excitatory postsynaptic current (mEPSC)]. Moreover, by comparing the current in untrained and trained animals, we analyzed the learning-induced plasticity (12, 13), and paired-pulse facilitation of AMPA receptor–mediated responses further allows determining the presynaptic glutamate-release probability (14, 15).

The nature of learning-induced pre- and postsynaptic plasticity is more complicated by the fact that learning also affects GABA_A_ receptor–mediated inhibitory synapses in CA1 pyramidal neurons (12, 16, 17). GABA_A_ receptors typically consist of 2 α subunits and 2 β subunits, together with either 1 γ or δ subunit (18). Pore opening allows Cl^−^ influx to induce a postsynaptic hyperpolarization upon GABA binding. Considering that each presynaptic vesicle contains ∼2500 molecules of GABA (19, 20), we also quantified miniature postsynaptic GABA_A_ receptor current induced by single-synaptic vesicle of GABA [miniature inhibitory postsynaptic current (mIPSC)]. Moreover, we compared the current in untrained and trained animals to analyze the learning-induced plasticity at GABA_A_ receptor–mediated synapses (12, 13). The paired-pulse depression of GABA_A_ receptor–mediated responses was also analyzed to determine the changes in presynaptic GABA-release probability (15, 21). By changing holding potential of the membrane, we took both mEPSC and mIPSC data sequentially from the same CA1 neuron and multidimensionally plotted the neurons to evaluate the synaptic diversity.

Genetic deficiency of GABA_A_ receptor β_3_ subunit severely impairs the contextual freezing response without affecting pain perception (22), and the phosphorylation in the cytoplasmic loop of β_3_ subunit (Ser^408−409^) is known to play an essential role for PKA, PKB, PKC, or Ca^2+^ and calmodulin–dependent protein kinase II–dependent plasticity (23). Becausee the phosphorylation is known to increase surface levels of GABA_A_ receptors containing β_3_ subunits in cultured neurons (24–27), we also examined the effect of learning as well as the temporal dynamics.

Pharmacological manipulation of the AMPA or GABA_A_ receptors in the CA1 suggested different roles of the receptors after training (12, 28–33). Microinjections of the AMPA receptor blocker [7-nitro-2,3-dioxo-1,4-dihydroquinoxaline-6-carbonitrile (CNQX)] into CA1 impairs inhibitory avoidance (IA) task training immediately (0–5 min) but the effects are lost 30–60 min after training (29, 30, 32), whereas GABA_A_ receptor blocker microinjection improves performance if performed immediately following training (28, 31–33). Although these studies suggest a critical period for plasticity immediately following training, the dynamic changes in learning-induced synaptic diversity are poorly understood. Here, we analyze the dynamic changes seen in learning-induced excitatory and inhibitory (*E*/*I*) synaptic function, pre- and postsynaptically. Learning rapidly strengthened both *E*/*I* synapses in various ways in individual CA1 neurons, producing a broad diversity of synaptic input across the CA1 neuronal population within 5 min after the training. Moreover, we quantified the diversity levels by calculating the self-entropy per single CA1 neuron.

## MATERIALS AND METHODS

### Animals

Male Sprague-Dawley rats (postnatal 4 wk of age) were obtained from Chiyoda Kaihatsu (Tokyo, Japan). Prior to the experiment, the rats were individually housed in plastic cages for a couple of days (40 × 25 × 25 cm) at a constant temperature (23 ± 1°C) under a 12-h light/dark cycle (lights on from 8 am to 8 pm) with *ad libitum* access to water and food (MF; Oriental Yeast, Tokyo, Japan). All animal housing and surgical procedures were approved by the Institutional Animal Care and Use Committee of Yamaguchi University Graduate School of Medicine and comply with the *Guide for the Care and Use of Laboratory Animals* [National Institutes of Health (NIH), Bethesda, MD, USA].

### IA task

**Figure 1:**
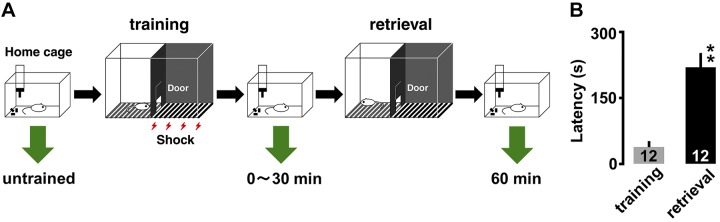
Diagram of experimental design and IA task. *A*) Rats were housed in a home cage but moved into the light box used for the task on the training day. A brief electrical foot-shock (2 s) was applied in the dark box in the shock cage. Brain slices were prepared at various time points of the training. *B*) Thirty minutes after the training, the rats consistently showed a longer latency before entering the dark side of the box. **P < 0.01 *vs.* training. Error bars indicate ± sem. The number of rats is shown at the bottom of each bar.

Hippocampus-dependent IA training procedures were previously described in refs. 6 and 12. The IA training apparatus (length, 33 cm; width, 58 cm; height, 33 cm) was a 2-chambered box consisting of a lighted safe side and a dark shock side separated by a trap door (**Fig. 1*A***). For training, rats were placed in the light side of the box facing a corner opposite the door. After the trap door was opened, the rats could enter the dark box at will. The latency before entering the novel dark box was measured as a behavioral parameter (latency before IA learning, Fig. 1*B*). Four seconds after the animals entered the dark side, we closed the door and applied a scrambled electrical foot-shock (2 s, 1.6 mA) *via* electrified steel rods in the floor of the box. The rats were kept in the dark compartment for 10 s before being returned to their home cage. The rats in the 0-min group were quickly euthanized with an overdose of pentobarbital within 1 min. Untrained control rats were not moved from their home cages and were injected with the same dose of anesthesia. The results of unpaired and walk-through controls were previously reported (12).

Thirty minutes after the procedure described above, the rats were placed in the light side. The latency before entering the dark box was measured as an indicator of learning performance (latency after IA learning).

### Slice patch-clamp

Acute brain slices were prepared as previously described in refs. 12 and 13. Detailed protocol of slice patch-clamp technique for analyzing learning-induced synaptic plasticity was also published with a short demonstration movie (34).

Rats were deeply anesthetized with pentobarbital at 0, 5, 10, 20, 30, or 60 min after the paired foot-shock. Then, the brains were quickly perfused with ice-cold dissection buffer (25.0 mM NaHCO_3_, 1.25 mM NaH_2_PO_4_, 2.5 mM KCl, 0.5 mM CaCl_2_, 7.0 mM MgCl_2_, 25.0 mM glucose, 90 mM choline chloride, 11.6 mM ascorbic acid, and 3.1 mM pyruvic acid) and gassed with 5% CO_2_ and 95% O_2_. Coronal brain slices (target CA1 area, AP -3.8 mm, DV 2.5 mm, LM ± 2.0 mm) were cut (350 µm; Leica vibratome; VT-1200; Leica Microsystems, Buffalo Grove, IL, USA) in dissection buffer and transferred to physiologic solution [22–25°C; 114.6 mM NaCl, 2.5 mM KCl, 26 mM NaHCO_3_, 1 mM NaH_2_PO_4_, 10 mM glucose, 4 mM MgCl_2_, and 4 mM CaCl_2_ (pH 7.4); gassed with 5% CO_2_ and 95% O_2_]. We maintained 3–4 brain slices for patch recordings based on the brain atlas by Paxinos and Watson (35). Glass electrodes were made with a horizontal puller (model P97; Sutter Instrument, Novato, CA, USA) and filled with a suitable solution. Whole-cell recordings were obtained from pyramidal neurons of the hippocampal CA1 layer, using an Axopatch-1D amplifier (Molecular Devices, Sunnyvale, CA, USA). Recordings were digitized using a Digidata 1440 AD board (Molecular Devices), recorded at 5 kHz, and analyzed offline with pClamp v.10.4 software (Molecular Devices).

### Miniature recordings

**Figure 2:**
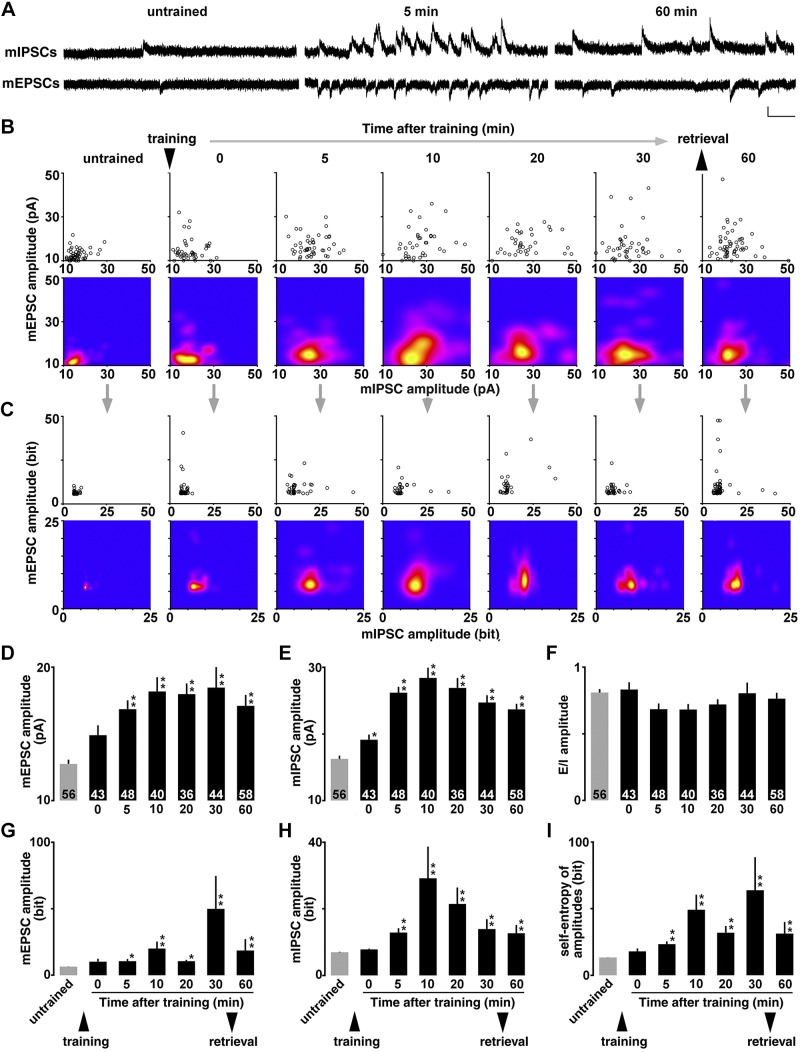
Temporal dynamics of the learning-induced diversity of mEPSC and mIPSC amplitudes and entropy analysis. *A*) Representative traces of mEPSCs and mIPSCs. We measured the mEPSCs at −60 mV and mIPSCs at 0 mV sequentially in the same CA1 pyramidal neuron in the presence of tetrodotoxin (0.5 µM). Vertical bar = 20 pA; horizontal bar = 50 ms. *B*) Plots of the means mEPSC and mIPSC amplitudes in each neuron (upper) and the visualized density by kernel analysis (lower). *C*) By calculating appearance probability of each dot, we plotted self-entropy (bit) of individual neurons (upper) and visualized the density by kernel analysis in untrained and trained rats (lower). *D*, *E*) Mean amplitude of mEPSCs (*D*) or mIPSCs (*E*) in all recorded neurons in untrained and trained rats. IA training increased mEPSC amplitude within 5 min, whereas it increased mIPSC amplitude within 1 min after the training (0 min). *F*) *E*/*I* balance of the mEPSC and mIPSC amplitude did not change after the training. *G*, *H*) The training also increased the self-entropy (bit) of excitatory (*G*) and inhibitory (*H*) synapses in CA1 neurons. *I*) Combined self-entropy (bit) of the mEPSC and mIPSC amplitudes in untrained and trained rats. The number of cells in each group is shown at the bottom of each bar. Error bars indicate + sem. *P < 0.05, **P < 0.01 *vs.* untrained. ▼ or ▲ indicate the timing of training or retrieval tests.

**Figure 3:**
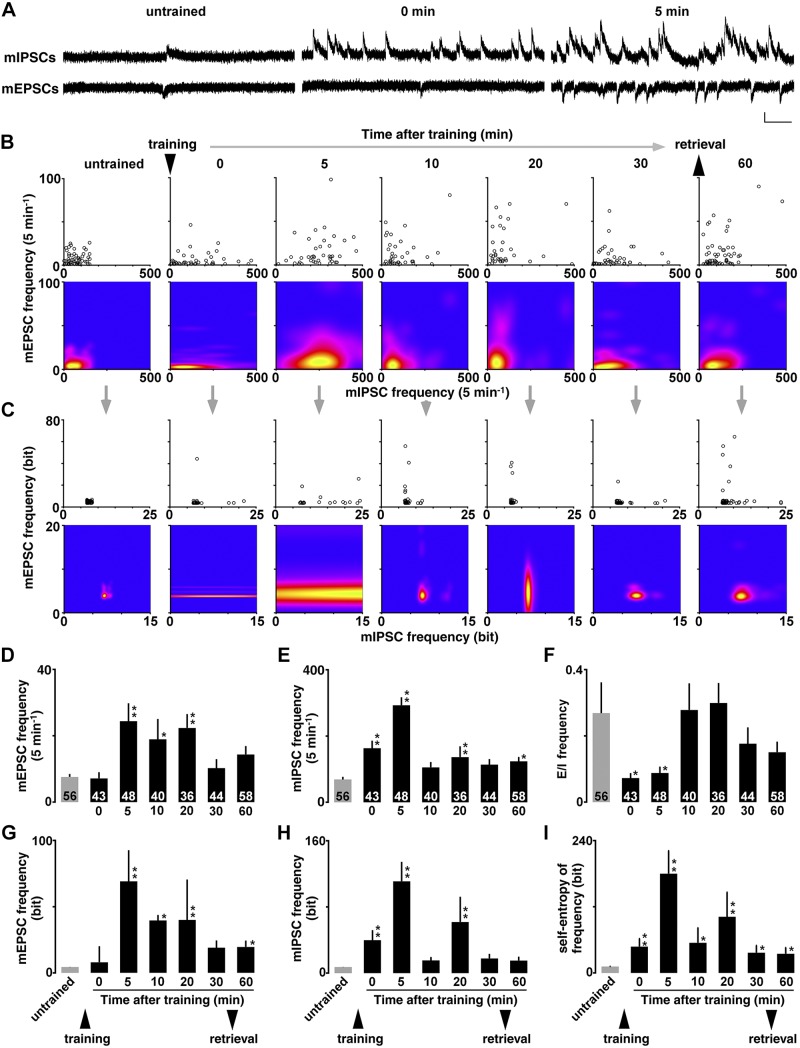
Temporal dynamics of the learning-induced diversity of mEPSC and mIPSC frequencies and entropy analysis. *A*) Representative traces of mEPSCs and mIPSCs. Vertical bar = 20 pA; horizontal bar = 50 ms. *B*) Plots of the means mEPSC and mIPSC frequency in each neuron (upper) and the visualized density by kernel analysis (lower). *C*) By calculating appearance probability of each dot, we plotted self-entropy (bit) of individual neurons (upper) and visualized the density by kernel analysis in untrained and trained rats (lower). *D*, *E*) Mean frequency of mEPSCs (*D*) or mIPSCs (*E*) in all recorded neurons in untrained and trained rats. IA training increased mEPSC frequency within 5 min, whereas it increased mIPSC frequency within 1 min after the training (0 min). *F*) *E*/*I* balance of the mEPSC and mIPSC frequency transiently decreased from 0 to 5 min after the training. *G*, *H*) The training also increased the self-entropy (bit) of excitatory (*G*) and inhibitory (*H*) synapses in CA1 neurons. *I*) Combined self-entropy (bit) of the mEPSC and mIPSC frequencies in untrained and trained rats. The number of cells in each group is shown at the bottom of each bar. Error bars indicate + sem. *P < 0.05, **P < 0.01 *vs.* untrained. ▼ or ▲ indicate the timing of training or retrieval tests.

For miniature recordings, we used a modified intracellular solution to adjust the reversal potential of the GABA_A_ receptor response [127.5 mM cesium methanesulfonate, 7.5 mM CsCl, 10 mM HEPES, 2.5 mM MgCl_2_, 4 mM Na_2_ATP, 0.4 mM Na_3_GTP, 10 mM sodium phosphocreatine, 0.6 mM EGTA (pH 7.25)]. Moreover, we added 0.5 µM tetrodotoxin (Wako Pure Chemicals, Osaka, Japan) to perfusate to block action potentials. The voltage was clamped at −60 mV for mEPSC recording and at 0 mV for mIPSC recording (Figs. 2*A* and 3*A*). We analyzed the frequency and amplitude of mEPSCs and mIPSCs above 10 pA.

We obtained 4 miniature parameters (mean mEPSC amplitude, mean mIPSC amplitude, mean mEPSC frequency, and mean mIPSC frequency) in individual CA1 pyramidal neurons. For graphic expression, the distribution was visualized 2-dimensionally in the R software environment (R Foundation for Statistical Computing, Vienna, Austria) (amplitude in **Fig. 2*B***; frequency in **Fig. 3*B***). To calculate *E*/*I* balance, the value of mEPSC frequency or amplitude was divided by corresponding value of mIPSC frequency or amplitude in each neuron. After recording, we confirmed that mEPSCs and mIPSCs were completely abolished by 10 µM CNQX (MilliporeSigma, Burlington, MA, USA) and 10 µM bicuculline methiodide (MilliporeSigma), respectively.

### Paired-pulse stimulation

To analyze presynaptic plasticity at excitatory synapses, we added 0.1 mM picrotoxin and 4 μM 2-chloroadenosine to the perfusate and performed paired-pulse stimulation at −60 mV. To analyze presynaptic plasticity at inhibitory synapses, we added 10 μM CNQX to the perfusate and performed paired-pulse stimulation at 0 mV. To evaluate the paired-pulse ratio from the EPSC or IPSC average, 50–100 sweeps were recorded with paired stimuli at 100-ms intervals. The ratio of the second amplitude to the first amplitude was calculated as the paired-pulse ratio.

### Western blotting

Western blotting was performed according to a previous study (13). Rats were deeply anesthetized with pentobarbital at 0, 5, or 30 min after the training. The brain was removed and incubated for 3 min in ice-cold buffer containing 0.32 M sucrose and 20 mM Tris–HCl (pH 7.5). Dissected hippocampal CA1 tissues were homogenized in 200 µl of buffer containing 50 mM Tris–HCl (pH 7.4), 0.5% Triton X-100, 0.5 M NaCl, 10 mM EDTA, 4 mM EGTA, 1 mM Na_3_VO_4_, 50 mM NaF, 40 mM sodium pyrophosphate, 1 mM protease inhibitor, and 1 mM DTT. Insoluble material was removed by a 10-min centrifugation at 15,000 rpm.

Samples containing equivalent amounts of protein based on the bicinchoninic acid analysis (Thermo Fisher Scientific, Waltham, MA, USA) were heated at 100°C for 3 min in Laemmli sample buffer and subjected to SDS-PAGE for 30 min at 200 V. Proteins were transferred to an immobilon PVDF membrane for 1 h at 100 V. Membranes were blocked for 1 h at room temperature in 50 mM Tris–HCl (pH 7.5), 150 mM NaCl, 0.1% Tween 20, and 5% skim milk. Then, the membranes were incubated overnight at 4°C with anti–GABA_A_ receptor β_3_ subunit (1:1000; Abcam, Cambridge, MA, USA), anti–phosphorylated GABA_A_ receptor β_3_ subunit (Ser^408−409^) (1:1000; Abgent, San Diego, CA, USA), or anti–β-tubulin (1:1000; BioLegend, San Diego, CA, USA). This step was followed by incubation with horseradish peroxidase–conjugated goat anti-rabbit IgG (1:5000; MilliporeSigma) for GABA_A_ receptor β_3_ subunit, phosphorylated GABA_A_ receptor β_3_ subunit, and β-tubulin. Bound antibodies were visualized using an ECL detection system (GE Healthcare, Chicago, IL, USA) and semiquantitatively analyzed using the ImageJ program (NIH).

### Self-entropy analysis

As we previously reported, standard spreadsheet software (Excel 2010; Microsoft, Redmond, WA, USA) was used to calculate the self-entropy per neuron (36). Although we obtained 4 miniature parameters (mean mEPSC amplitude, mean mIPSC amplitude, mean mEPSC frequency, and mean mIPSC frequency) in individual CA1 pyramidal neurons, we determined the distribution of appearance probability of each miniature parameter using 1-dimensional kernel density analysis. *X*_1_, *X*_2_, . . ., *X_n_* denotes a sample of size *n* from real observations. The kernel density estimate of *P* at the point *x* is given by
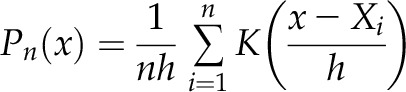
where *K* is a smooth function called the gaussian kernel function and *h* > 0 is the smoothing bandwidth that controls the amount of smoothing. We chose Silverman’s reference bandwidth or Silverman’s rule of thumb (37, 38). It is given by:
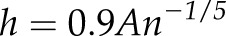
where *A* = min (sd, interquartile range 1.34). By normalizing integral value in untrained controls, we found the distribution of appearance probability at any point. Then, we calculated the appearance probability at selected points. All data points for probability in untrained and trained rats were converted to self-entropy (bit) using the Shannon entropy concept defined from the information theory (39).

In the spreadsheet software, the data of 4 miniature parameters were summarized in 4 different sheets, and we obtained the bandwidth (*h*) of individual parameter in untrained group using a formula [=0.9 STDEV (neuron 1, neuron 2, neuron *N*) /COUNT (neuron 1, neuron 2, neuron *N*)^*1/5*^]. Then, using the data of the untrained group, we calculated the distribution of appearance probability as follows:

1. Probability distribution of first data of a parameter (neuron 1) was calculated using a formula [=EXP(-(((data of neuron 1 − any point)/*h*)^*2/2*^))/SQRT (2 × PI())].
2. Also, probability distribution of second data of the parameter (neuron 2) was calculated using the formula [=EXP(-(((data of neuron 2 − any point)/*h*)^*2/2*^)) /SQRT(2 × PI)].
3. Similarly, probability distribution of *N* data of the parameter (neuron *N*) was calculated using the formula [=EXP(-(((data of neuron *N* − any point)/*h*)^*2/2*^)) /SQRT(2 × PI)].
4. Sum all probability distribution from neuron 1 to *N*, and the integral value was normalized to 1.

Based on the probability distribution, we calculated individual appearance probability of all recorded neurons. Then, the appearance probability of the neuron was converted to the self-entropy using Shannon's formula [=−LOG (appearance probability of the neuron, 2)]. For graphic expression, we visualized the self-entropy distribution by 2-dimensional kernel analysis in the R software environment (Figs. 2*C* and 3*C*).

### Statistical analysis

We used the paired Student’s *t* test to analyze the latency. The data of mEPSC, mIPSC, self-entropy, and protein levels were analyzed using 1-way factorial ANOVA in which the between-group factors were the individual time points. The Shapiro-Wilk test and *F* test were used for normality and equality of variance, respectively. Because the self-entropy data had large variations within a group, we performed log (1 + *x*) transformation prior to the analysis (40). A value of *P* < 0.05 was considered significant.

## RESULTS

### The performance of IA task

To investigate learning-induced synaptic modification in the hippocampus, we used the IA task (Fig. 1*A*). In this paradigm, rats were allowed to cross from a light box to a dark box, where an electric foot-shock (1.6 mA, 2 s) was delivered. Half an hour after the task, we measured the latency in the illuminated box as contextual learning performance. Figure 1*B* shows the latency in the training session and the retrieval test. The rats consistently showed longer latency in the retrieval test than in the training session (Fig. 1*B*; *t*_11_ = 5.746; *P* < 0.0001).

### Miniature postsynaptic currents

To analyze the learning-dependent synaptic plasticity, we recorded mEPSC or mIPSC in the presence of 0.5 µM tetrodotoxin on the dorsal hippocampus (Fig. 2*A*). By changing the membrane potential, we sequentially recorded mEPSCs (at −60 mV) and mIPSCs (at 0 mV) from the same neuron, as previously reported in refs. 12 and 13. We confirmed that the mEPSC and mIPSC events were clearly blocked by the bath treatment of an AMPA receptor blocker (CNQX) or GABA_A_ receptor blocker (bicuculline). The postsynaptic currents are thought to correspond to the response elicited by a single vesicle of glutamate or GABA (41). In contrast, the number of synapses affects the frequency of events.

We found cell-specific mean AMPA receptor–mediated excitatory currents *vs.* GABA_A_ receptor–mediated inhibitory currents in each neuron and plotted them 2-dimensionally [amplitude in Fig. 2*B* (upper); frequency in Fig. 3*B* (upper)]. Although untrained rats exhibited a narrow distribution, trained rats had a broad distribution suggesting a diversity of synaptic currents in CA1 pyramidal neurons [amplitude in Fig. 2*B* (lower); frequency in Fig. 3*B* (lower)]. In the mEPSCs, 1-way ANOVA revealed significant temporal change [amplitude in Fig. 2*D*, *F*_(6, 318)_ = 5.448, *P* < 0.0001; frequency in Fig. 3*D*, *F*_(6, 318)_ = 3.738, *P* = 0.0013]. Compared with untrained control, *post hoc* analysis further showed a significant effect from 5 to 60 min after the training (Fig. 2*D*). Also in the mIPSCs, 1-way ANOVA revealed significant temporal change [amplitude in Fig. 2*E*, *F*_(6, 318)_ = 20.296, *P* < 0.0001; frequency in Fig. 3*E*, *F*_(6, 318)_ = 19.492; *P* < 0.0001]. *Post hoc* analysis further showed a significant effect from 0 to 60 min after the training (amplitude in Fig. 2*E*; frequency in Fig. 3*E*). These results suggest rapid postsynaptic strengthening at both *E*/*I* synapses within 5 min after the training.

Continuous plasticity in the sliced condition may affect the synaptic functions. To rule out this possibility, we examined the change of mEPSC and mIPSC amplitude or frequency along with the elapsed time of the slice in the interface chamber. To this end, we recorded mEPSCs and mIPSCs from CA1 pyramidal neurons 1–10 h after the slice preparation, showing no correlation between the mEPSC and mIPSC parameters and the elapsed time of the slice (mEPSC amplitude, *R*^2^ = 0.003; mEPSC frequency, *R*^2^ = 0.083; mIPSC amplitude, *R*^2^ = 0.080; mEPSC frequency, *R*^2^ = 0.134; *N* = 75).

### *E*/*I* balance

To calculate the balance of *E*/*I* inputs, mean mEPSC amplitude was divided by mean mIPSC amplitude and mean mEPSC frequency was divided by the mean mIPSC frequency in each neuron. In the *E*/*I* balance of the amplitudes, the main effect of training [*F*_(6, 318)_ = 1.570; *P* = 0.16] was not significant after the training (Fig. 2*F*). Conversely, in the *E*/*I* balance of miniature frequency, the main effect of training was significant after the training [Fig. 3*F*, *F*_(6, 318)_ = 2.371; *P* = 0.0296]. *Post hoc* analysis further showed a significant decrease from 0 to 5 min after the training, suggesting faster postsynaptic plasticity at the inhibitory synapses than that at excitatory synapses (Fig. 3*F*).

### Self-entropy of mEPSC and mIPSC amplitude

Based on the information theory of Shannon (39), we calculated the appearance probability of the mean amplitudes of mEPSCs and mIPSCs. First, we found the distribution of appearance probability in untrained controls (Fig. 2*B*, left) and then we analyzed cell-specific appearance probability of all recorded neurons one by one (Fig. 2*C*, upper panels). Each probability of a single neuron was calculated as the self-entropy and plotted 2-dimensionally. For example, a point with a high appearance probability (around the mean level of mEPSC and mIPSC amplitude) indicated low self-entropy, whereas a point with very rare probability (a deviated point of mEPSC and mIPSC amplitude) indicated high self-entropy.

Two-dimensional kernel analysis visualized the density (Fig. 2*C*, lower panels). IA training clearly diversified the amount of information per neuron and sustained. For the mEPSC amplitude, the results are statistically summarized in Fig. 2*G*. Self-entropy in the mEPSC amplitude exhibited a significant temporal change [*F*_(6, 318)_ = 13.456; *P* < 0.0001]. *Post hoc* analysis further showed a significant effect from 5 to 60 min after the training compared with untrained control. Similarly, self-entropy in the mIPSC amplitude exhibited a significant temporal change [*F*_(6, 318)_ = 15.057; *P* < 0.0001; Fig. 2*H*], and *post hoc* showed a significant effect from 5 to 60 min after the training. Combined self-entropy (bit) at both synapses also increased within 5 min after the training [*F*_(6, 318)_ = 8.565, *P* < 0.0001; Fig. 2*I*].

### Self-entropy of mEPSC and mIPSC frequency

For mEPSC and mIPSC frequency, we found the distribution of appearance probability in untrained controls (Fig. 3*B*, left), and then we analyzed the appearance probability of all recorded neurons one by one. We found cell-specific self-entropy in all recorded neurons, showing different self-entropy from each other (Fig. 3*C*, upper panels).

Two-dimensional kernel analysis visualized the density (Fig. 3*C*, lower panels). IA training diversified the amount of information per neuron and sustained. For the mEPSC frequency, the results are statistically summarized in Fig. 3*G*. Self-entropy in the mEPSC frequency exhibited a significant temporal change [*F*_(6, 318)_ = 3.694; *P* = 0.0015]. *Post hoc* analysis further showed a significant effect from 5, 10, 20, and 60 min after the training compared with untrained control. In contrast, self-entropy in the mIPSC frequency increased quite rapidly [*F*_(6, 318)_ = 20.938; *P* < 0.0001; Fig. 3*H*], showing a long horizontal kernel distribution at 0 and 5 min after the training (Fig. 3*C*, lower). Significant effect was observed at 0, 5, and 20 min after the training. Moreover, combined self-entropy (bit) at both synapses significantly increased within 1 min after the training [*F*_(6, 318)_ = 15.511, *P* < 0.0001; Fig. 3*I*].

### Presynaptic glutamate and GABA release

**Figure 4:**
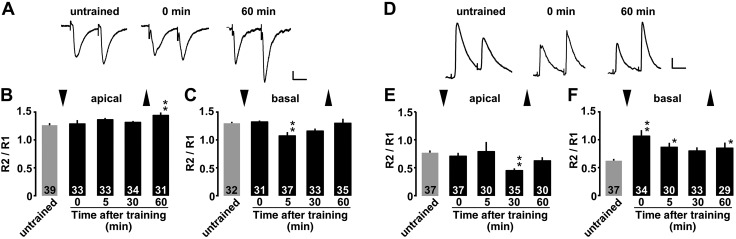
Presynaptic glutamate and GABA release probability. *A*) Representative traces of AMPA receptor–mediated paired-pulse responses at apical dendrites of CA1. *B*, *C*) Mean ratio at the apical (*B*) or basal dendrites (*C*) in untrained and trained rats. *D*) Representative traces of GABA_A_ receptor–mediated paired-pulse response at basal dendrites of CA1. *E*, *F*) Mean ratio at the apical (*E*) or basal dendrites (*F*) in untrained and trained rats. Vertical bars = 20 pA; horizontal bars = 50 ms. *P < 0.05, **P < 0.01 *vs.* untrained. The number of neurons is shown at the bottom of each bar. Error bars indicate ± sem. ▼ and ▲ indicate the timing of training and retrieval tests, respectively.

To analyze presynaptic plasticity, we examined the paired-pulse ratio after the training. At the excitatory synapses in the apical dendrite, the paired-pulse ratio for evoked EPSCs was significantly increased at 60 min after training, suggesting a delayed decrease in presynaptic glutamate release probability [**Fig. 4*A, B***; *F*_(4, 172)_ = 2.588; *P* = 0.0386, 1-way factorial ANOVA]. At the excitatory synapses in the basal dendrite, the paired-pulse ratio for evoked EPSCs was significantly decreased at 5 min after the training, suggesting a transient increase in presynaptic glutamate release probability [Fig. 4*C*; *F*_(4, 163)_ = 3.994; *P* = 0.0041, 1-way factorial ANOVA]. Conversely, at the inhibitory synapses in the apical dendrite, the paired-pulse ratio for evoked IPSCs was significantly decreased at 30 min after the training, suggesting a transient increase in the presynaptic GABA release probability [Fig. 4*E*; *F*_(4, 164)_ = 2.818; *P* = 0.0269, 1-way factorial ANOVA]. At the inhibitory synapses in the basal dendrite, the paired-pulse ratio for evoked IPSCs was significantly increased at 0, 5, and 60 min after the training, suggesting a rapid decrease in the presynaptic GABA release probability within 1 min after training [Fig. 4*D, F*; *F*_(4, 158)_ = 4.389; *P* = 0.0022, 1-way factorial ANOVA].

### Phosphorylation of GABA_A_ receptor subunits

**Figure 5:**
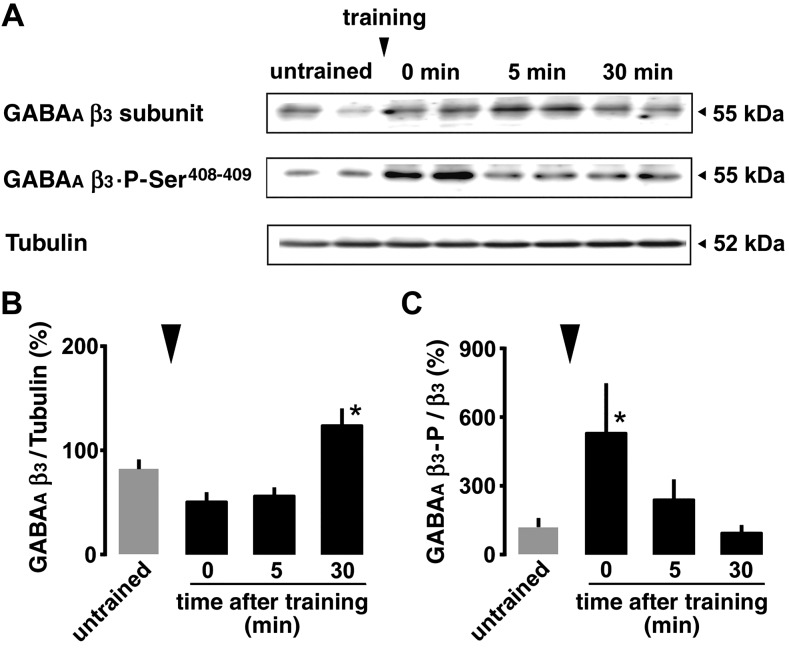
Rapid Ser^408−409^ phosphorylation of GABA_A_ receptor β_3_ subunit. *A*) Whole-cell fractions of dissected dorsal CA1 were analyzed. Molecular masses of standards are indicated on the right. *B*) Total GABA_A_ receptor β_3_ subunit was normalized to total β-tubulin, showing an increase in the subunit 30 min after the training. *C*) The phosphorylation-specific signals of Ser^408−409^ were normalized to the total GABA_A_ receptor β_3_ subunit. IA training increased Ser^408−409^ phosphorylation within 1 min after the training (0 min). *P < 0.05, **P < 0.01 *vs.* untrained; *n* = 9 in all groups. Error bars indicate ± sem. ▼ indicates the timing of training.

To analyze phosphorylation of the receptors using Western blot, we trimmed dorsal hippocampal CA1 tissue and extracted the whole-cell fractions (**Fig. 5*A***). GABA_A_ receptor β_3_ subunit levels were significantly increased at 30 min after the training [Fig. 5*B*; *F*_(4, 40)_ = 10.237; *P* = 0.0001, 1-way factorial ANOVA]. Moreover, Ser^408−409^ phosphorylation of the β_3_ subunit was significantly increased within 1 min after the training [Fig. 5*C*; *F*_(4, 40)_ = 3.117; *P* = 0.0253, 1-way factorial ANOVA].

## DISCUSSION

### Rapid plasticity at excitatory synapses

Although rapid plasticity of excitatory CA1 synapses is considered as an initial step of memory encoding rather than retrieval (42), conclusive evidence for the dynamic change of synaptic current is still lacking. Here we found a rapid increase in mEPSC amplitude within 5 min after IA training, showing that memory encoding rather than retrieval strengthens AMPA receptor–mediated excitatory synapses. Using fluctuation analysis of CA1 pyramidal neurons, we recently confirmed that the training increased postsynaptic number of AMPA receptor channels without changing cation current per single channel (36). As to the causal relationship between learning and the plasticity, we previously reported the bilateral gene expression of GluA1-containing AMPA receptor delivery blockers in the CA1 neurons impairs IA learning (6). Moreover, a chromophore-assisted light-inactivation technique demonstrated that optical inactivation of synaptic AMPA receptors can erase acquired memory (43). These results showed that newly delivered GluA1-containing AMPA receptors contribute to form contextual memory.

Paired-pulse analysis further revealed the presynaptic glutamate plasticity after the training. The decrease in the paired-pulse ratio at 5 min after the training suggests transient increase in presynaptic glutamate release within 5 min after the training. Because mEPSC frequency is used as an indicator for evoked release (44–46) or the number of functional synapses (47, 48), both pre- and postplasticity may contribute to the increase in mEPSC frequency at 5 min after the training. Although the immobilization of postsynaptic AMPA receptors can also decrease the paired-pulse ratio (49), bilateral CA1 microinjections of the AMPA receptor blocker (CNQX) seem to impair the IA learning immediately (0–5 min) but not at 30–60 min after the training (29, 30, 32). Optogenetic approach is a powerful technique to investigate spine-specific presynaptic plasticity after contextual learning; glutamate-release probability at the synapses between CA3-engram and CA1-engram cells was significantly greater than that of other pair types of synapses (50). These findings together with the present results support the notion that contextual learning requires the presynaptic acute glutamate release soon after the training.

### Rapid plasticity at inhibitory synapses

Conversely, the plasticity at inhibitory synapses seems to be task dependent and region specific (12, 13, 16). As to hippocampal-dependent contextual learning, IA training clearly increased the mIPSC amplitudes, suggesting a postsynaptic strengthening of GABA_A_ receptor–mediated plasticity (12). Also, the mIPSC frequency was rapidly increased without increase in GABA release probability, suggesting rapid activation of inhibitory silent or subthreshold synapses to increase the number of over-threshold synapses. It is possible that many mIPSC events are small and below the level of detection threshold (<10 pA) and increased postsynaptic responses may increase the amplitude of these small events above the level of detection (>10 pA), resulting in an apparent increase in mIPSC frequency. Although the mechanism at GABAergic synapses is still unclear, it was well-described regarding the postsynaptic function of GluA3 containing AMPA receptors in the hippocampal CA1 (51). Moreover, we further found a rapid increase in mIPSC amplitude immediately after the training, indicating that the memory encoding rather than the retrieval strengthens the GABA_A_ receptor–mediated inhibitory synapses. This is the first report showing a rapid phosphorylation of the Ser^408−409^ GABA_A_ receptor β_3_ subunit within 1 min after the training, the sites of which are necessary to attenuate clathrin-dependent endocytosis of the synaptic receptors increasing both amplitude and frequency of mIPSCs in cultured neurons (52).

A possible causal relationship between the GABAergic plasticity and learning has been previously reported. Not only the genetic deficiency of GABA_A_ receptor β_3_ subunit but also the prevention of GABA_A_ receptor–mediated plasticity in CA1 impairs the contextual learning (12, 22). Optogenetic manipulation of CA1 neurons further proved the timing-specific causal relationship between the GABAergic inputs and the learning; optic inactivation of dendrite-targeting CA1 interneurons during aversive stimuli was sufficient to prevent fear learning (17). In a preliminary study, we found that microinjections of an interference peptide in Ser^408−409^ phosphorylation into the CA1 successfully blocked the training-induced strengthening of mIPSCs. Moreover, bilateral microinjections of the peptide resulted in a drastic decrease in IA task-learning performance, suggesting further causal relationship between the learning and the Ser^408−409^ phosphorylation of the GABA_A_ β_3_ subunit.

Questions arise as to how the training can increase GABA_A_ receptor–mediated currents so rapidly. Mobility of GABA_A_ receptors may be closely associated with the issue, because the removal from the postsynaptic membrane or lateral diffusion decreases the synaptic GABAergic current (53–55). Recent single-particle tracking analysis further demonstrated quick diffusion of a single GABA_A_ receptor (0.07 µm^2^/s) in cultured hippocampal neurons; it can move rapidly between the 2 different synapses within a few hundred milliseconds to a few seconds. Surprisingly, abundant GABA_A_ receptors heterosynaptically locate at glutamatergic synapses, playing a key role in the stimulus-dependent rapid changes in the postsynaptic number of receptors (56). Because single CA1 pyramidal neuron possesses around 30,000 excitatory and 1700 inhibitory synapses (57), learning may rapidly recruit the heterosynaptic GABA_A_ receptors to strengthen the inhibitory synapses.

Once the receptor reaches the postsynaptic region through lateral diffusion (53, 54), gephyrin seems to stabilize the synaptic receptors (23, 58). Gephyrin can bind major subunits of GABA_A_ receptors (α_1–3_ and β_2–3_) (59), and the prevention decreases mIPSC amplitudes (60). Because the phosphorylation of Ser^408−409^ GABA_A_ receptor β_3_ subunit is known to prevent clathrin adaptor protein 2–mediated GABA_A_ receptor internalization, the training-induced Ser^408−409^ phosphorylation may help to stabilize the surface receptors (61–63). Although the training-induced Ser^408−409^ phosphorylation is rapid and transient, gephyrin may contribute to sustaining large mIPSC amplitude. Finally, using fluctuation analysis of CA1 pyramidal neurons, we recently confirmed that the training increased the postsynaptic number of GABA_A_ receptor channels without changing Cl^−^ current per single channel (36).

### Overall significance of temporal dynamics

An early-phase long-term potentiation (<1 h) is thought to arise from rapid changes in the functional status of preexisting synapses, which may include conversion of synapses from silent state to an active one (47) and increase in the release probability of presynaptic vesicles (45, 46). Because the training did not increase the release probability except some time points (Fig. 4; glutamate at 5 min and GABA at 30 min), it may increase the number of functional synapses drastically. Considering the peak levels of mEPSC and mIPSC frequency, the number of over-threshold synapses (>10 pA) may increase up to 2–4 times greater than the pretraining levels and then decreased gradually (excitatory synapses, Fig. 3*D*; inhibitory synapse, Fig. 3*E*).

Temporal dynamics of mEPSCs and mIPSCs further revealed a time lag of synaptic plasticity. IA training rapidly increased both mIPSC amplitude and frequency within 1 min, whereas the training enhanced both mEPSC amplitude and frequency within 5 min. Moreover, the time lag transiently reduced *E*/*I* balance of the mEPSC and mIPSC frequency but returned to the pretraining level within 10 min (Fig. 3*F*). The quicker plasticity at inhibitory synapses may contribute to reduce seizure vulnerability on the remodeling of hippocampal networks (64).

The *E*/*I* imbalance may be caused by training-induced spike bursts of CA1 neurons, because optogenetically-induced spike burst reduces *E*/*I* balance in CA1 pyramidal neurons (65). Then, concomitant calcium influx may induce the specific phosphorylation of GluA1 and GABA_A_ receptor β_3_ subunits. Abundant extrasynaptic membrane GABA_A_ receptors of CA1 pyramidal neurons (66) and the rapid motility (53) may enable rapid activation of GABA_A_ receptor–mediated synapses. In contrast, it is known to take at least 5 min for long-term potentiation induction, because GluA1-containing AMPA receptors are inserted at the plasma membrane and move laterally to the excitatory synapses (67, 68).

### Quantification of diversity

Shannon’s information theory was applied to the recent studies on learning and memory (69, 70). Although the induction of long-term potentiation expands synaptic information storage capacity in hippocampal dentate gyrus neurons *in vivo* (70), it is still unknown whether the learning affects the information content of CA1 neurons. Here, we calculated the self-entropy of each CA1 neuron to quantify the learning-induced synaptic diversity. Each CA1 neuron had a different self-entropy that was clearly increased after the training (Figs. 2*C* and 3*C*). Because bilateral blockade of the synaptic diversity clearly impaired the learning performance (12, 71), we hypothesized that the increased self-entropy may code a piece of experienced information after training. Considering the total number of pyramidal neurons in rat dorsal CA1 (72), the self-entropy in untrained condition (5.2 × 10^6^ bits) would increase up to 42.1 × 10^6^ bits at 10 min after the training. In any case, the analysis may be a useful approach to quantify the learning-induced synaptic diversity at the entropy level.

### Temporal dynamics and cognitive disorders

Synaptic dysfunction is well-correlated with cognitive decline in Alzheimer’s disease (73). Amyloid β peptide 1–42 (Aβ_42_) is well-known as a major causative agent (74–77), and long-term exposure to Aβ_42_ (1–3 d) impairs the AMPA receptor trafficking by reducing synaptic distribution of Ca^2+^ and calmodulin–dependent protein kinase II in cultured pyramidal neurons (78). In contrast, the effect of soluble oligomeric assemblies of Aβ_42_ is more rapid, decreasing surface level of AMPA receptors within 30 min (79). Although less is known about the toxic effect at inhibitory synapses, Aβ_42_ specifically binds to nicotinic α_7_ receptors (80), impairing the learning-induced plasticity at the GABA_A_ receptor–mediated inhibitory synapses (12, 81). Bath application of Aβ_42_ weakens GABA_A_ receptor–mediated synaptic currents within 10 min (82), whereas it directly blocks the nicotinic α_7_ receptor–mediated cholinergic response within 3 min (83). Understanding the dynamic changes occurring during learning-promoted plasticity would be necessary to identify a failure point in cognitive disorders.

## Abbreviations

Aβ_42_: amyloid β peptide 1–42
AMPA: α-amino-3-hydroxyl-5-methyl-4-isoxazole-propionate
CA1: cornu ammonis 1
CNQX: 7-nitro-2,3-dioxo-1,4-dihydroquinoxaline-6-carbonitrile
*E/I*: excitatory and inhibitory
EPSC: excitatory postsynaptic current
GluA: glutamate receptor subunit A
IA: inhibitory avoidance
IPSC: inhibitory postsynaptic current
mEPSC: miniature EPSC
mIPSC: miniature IPSC
